# Online Collaborative Perception of Full Bridge Deck Driving Visual of Far Blind Area on Suspension Bridge during Vortex-Induced Vibration

**DOI:** 10.3390/s24061934

**Published:** 2024-03-18

**Authors:** Danhui Dan, Gang Zeng, Xuewen Yu

**Affiliations:** School of Civil Engineering, Tongji University, Shanghai 200092, China; 2032305@tongji.edu.cn (G.Z.); xuewen_yu@tongji.edu.cn (X.Y.)

**Keywords:** vertical vortex-induced vibration, far blind area, driving safety, real-time dynamic configuration, maximum height of blind area, effective sight distance

## Abstract

During a vertical vortex-induced vibration (VVIV), an undulating bridge deck will affect drivers’ sightlines, causing the phenomenon of drifting and changes in the far blind area, thus presenting a potential threat to driving safety. Consequently, to ensure the safety of driving on a suspension bridge deck under VVIV, it is necessary to perceive the far blind spot caused by the occlusion of the driving sightlines under this condition, and to establish an online perception and evaluation mechanism for driving safety. With a long-span suspension bridge experiencing VVIV as the engineering background, this paper utilizes the acceleration integration algorithm and the sine function fitting method to achieve the online perception of real-time dynamic configurations of the main girder. Then, based on the configurations, the maximum height of the driver’s far blind area and effective sight distance are calculated accordingly, and the impact of different driving conditions on them is discussed. The proposed technical framework for driving safety perception in far blind spots is feasible, as it can achieve real-time estimation of the maximum height and effective distance of the far blind area, thereby providing technical support for bridge–vehicle–human collaborative perception and traffic control during vortex-induced vibration.

## 1. Introduction

Vortex-induced vibration (VIV) [1,2] is a common wind-induced vibration phenomenon occurring on various types of bridges generated at a lower wind speed, especially in long-span suspension bridges [3,4,5,6,7,8,9]. When the frequency of vortex shedding is close to one of the bridge’s natural frequencies, VIV will happen, and the vibration amplitude of the beam will continue to increase until it reaches stability. VIVs may happen in torsional or vertical directions, with vertical VIVs (VVIVs) being considered in this paper.

When a bridge experiences VIV, especially high-order VIV, the bridge deck will rise and fall, blocking the driver’s line of sight and causing psychological panic, thereby threatening driving safety. In particular, the instantaneous change in the far blind area may exacerbate the driver’s psychological experience of bridge vibration, causing them to associate the sightline disturbance and bridge vibration with the unsafe design of bridges, resulting in adverse social impacts on bridges’ operation. Larsen et al. [10] evaluated the VVIV performance of the Great Belt East Bridge in Denmark and pointed out that the raised bridge deck would block the driver’s vision to a certain extent, posing both a distraction to the driver and a possible hazard to traffic safety. Recently, VIVs also occurred on the Xihoumen Bridge and were recorded by the health monitoring system [11,12,13]. It can also be found from the surveillance video that the distant sight is blocked when driving during VIV [14].

To ensure the safety of drivers on vertical curves, Chinese Highway Engineering Technical Standards (JTG B01-2014) [15] specify the required parking sight distances at different design speeds for expressways and first-class highways, as well as the corresponding vertical curve radius and length limits. As for VVIV, however, it is insufficient to stipulate that the vertical curve formed by the vortex vibration meets the parking sight distance; a strict requirement that the vertical curve does not interfere with the driver’s sightline is more reasonable. According to the needs of driving sight distance, Chen et al. [16] investigated the vertical bending vortex vibration limit of long-span bridges considering the impact on driving safety. Chen [17] developed the calculation model of a vortex vibration limit that weighed driving safety and devised an iterative method to solve it. Compared to the technique used in the norm and its limit value, the obtained vortex vibration limit value was lower than the normative value. Based on monitoring data, Cao et al. [14] assessed the driving comfort and safety of suspension bridges experiencing VIV. Zhu et al. [18] developed a mathematical formula for computing the driver’s far blind zone on a bridge deck suffering from VIV and analyzed the impact of vehicle factors on the duration time of far blind spots and its proportion; however, the calculation of the maximum height of the far blind zone and the vehicle displacement are not accurate enough, which may adversely affect the evaluation of driving safety. Therefore, it is necessary to investigate a more precise calculation method for the far blind zone analysis of suspension bridges suffering VVIV.

Various codes and studies currently assess bridge VIV performance from the perspectives of VIV limit, driving safety, and the fatigue of structural components [19,20,21,22]. However, few studies have focused on the online perception of the far and near blind areas and the calculation of related parameters. This paper explores how to perceive and evaluate the far blind spot of driving vision based on the identification of a dynamic configuration of the main beam suffering from VVIV, and investigates the impact of different driving conditions on the far blind spot. The main innovations of this paper are as follows: a new definition of the effective sightline of the driver during VVIV is given; an accurate calculation method for the maximum height of the far blind zone and effective sight distance of the driver during VVIV of the main girder is established; and the influence law of different vehicle models, vehicle speeds, and the time of a vehicle entering the bridge on the maximum height of the blind area and the effective sight distance. This paper is a companion piece to the authors’ previous work [23], which studied the online perception of the front blind area of vehicles on a full bridge during VVIV.

The research in this paper contributes to forming a real-time assessment and decision-making mechanism for driving safety during VVIV. It then provides a specific application scenario of vehicle, road, and bridge collaborative perception for intelligent transportation. Intelligent vehicle perception hardware, such as cameras and radar, is based on biological senses, and they are mounted on the vehicle side, inevitably creating blind spots. Regardless of how intelligent a system may be, it is limited to making rapid and precise decisions within the visual field. In a high-speed smart car, for instance, if an object suddenly appears in the blind area of vision, it is difficult for the system to avoid an accident due to inertia, even if the system decides to brake suddenly or continue driving. To address this problem, roadside infrastructures are digitized by means of digital twins, and road environment information is transmitted in real time. The road information is expected to assist vehicles in discovering things beyond their vision and in processing data, and to communicate with vehicles through 5G to achieve intelligent transportation.

## 2. Maximum Height of Blind Spot and Effective Sight Distance of Driving on Bridge Deck during VVIV: Basic Concepts and Perception Routes

This section introduces the basic concepts of the maximum height of the blind spot and the effective sight distance, followed by the technical framework for the online estimation of them during VVIV.

### 2.1. Maximum Height of Blind Area and Effective Sight Distance of Driving on Bridge Deck during VVIV

**Figure 1:**
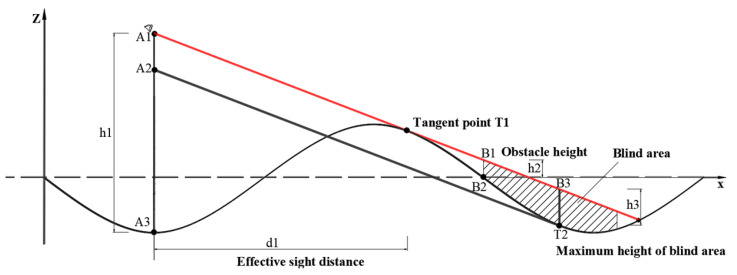
Effective sight distance of the driver and maximum height of blind area when the driver is at position A3.

When the bridge experiences VVIV, the vertical curve of the deck is related to the VIV mode and changes periodically with the vibration. Generally, in a vibration mode with a combination of several crests and troughs, the driver’s sightline is blocked by the crests ahead when the vehicle is in a trough. Figure 1 takes the VIV mode of three half-waves as an example. When the vehicle is located in the maximum trough of the mode, the far blind spot caused by the occlusion of the driving sightlines is illustrated.

**Figure 2:**
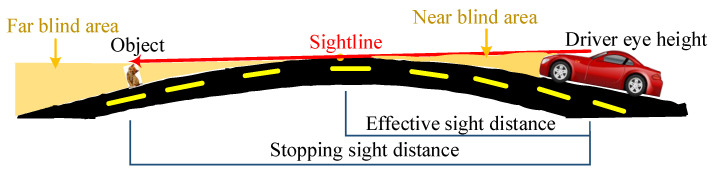
Diagram of effective and stopping sight distance when a driver goes uphill.

The mode shape of the bridge is assumed to be a simple harmonic form, and the distance between two adjacent stagnation points is L1. As shown in Figure 2, A3 represents the vehicle’s current position on the main beam; A1 is the driver’s binocular position; h1 represents the driver’s eye height. There is a tangent line from the driver’s eye A1 to the main beam. The tangent point is T1. Suppose there is an obstacle at B2, and the obstacle’s height is h2, then the obstacle will be invisible within a certain distance from B2. In the vehicle’s forward direction, there is a visual blind spot in the adjacent wave valley area, and objects with a height less than h2 within this blind spot are invisible to the driver. At this time, the maximum height of the visual blind zone is the maximum height of blind area.

The road sight distance mainly includes stopping sight distance, overtaking sight distance, passing vehicle sight distance, recognition sight distance, etc. Stopping sight distance is the shortest distance the driver must travel before reaching the obstacle from the moment the driver first observes the obstacle in front of the vehicle at a certain speed. In the inspection of the parking sight distance, the driver’s viewpoint height is 1.2 m for the parking sight distance of a passenger car, and the height of the apex of the obstacle on the road ahead of the viewpoint is 0.1 m [15]. One criterion for judging this is that the blind spot cannot exceed 10 cm in height.

When the bridge suffers from VVIV, the driver sees that road markings disappear before his eyes, causing him to psychologically panic, which affects his driving safety. Therefore, it is necessary to perceive the distance in real time. During normal driving, effective sight distance is the distance over which the driver can continuously see road markings within the lane in front on the highway. The distance here refers to the distance along the center of the lane. Effective sight distance, as shown in Figure 2, corresponds to the horizontal distance from the eye to the starting point of the blind spot, that is, the horizontal distance from A2 to the tangent point T1 of the sightline and the curve of the road surface.

### 2.2. Real-Time Online Perception Framework for Maximum Height of Blind Spot and Effective Sight Distance

**Figure 3:**
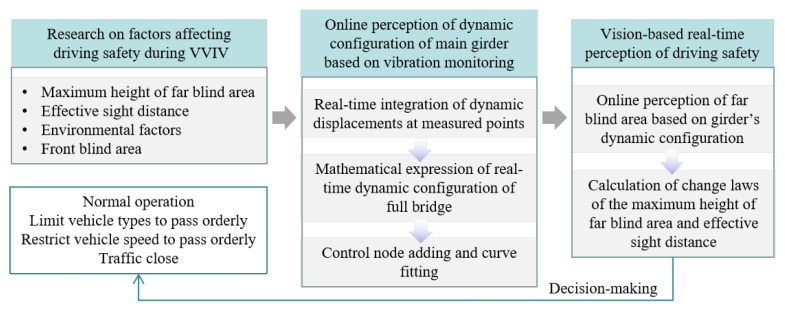
Technical route of online perception of the changing law of the driving far blind area on the full-bridge deck based on the real-time dynamic configuration of the main girder.

Based on real-time dynamic configuration monitoring of the main girder, this paper presents a real-time online framework for the driver’s visual blind spots on the whole bridge deck. The framework uses real-time dynamic configurations of the main beam based on monitoring vibration data to establish a method to determine the maximum height of the blind area and the effective sight distance of the driver’s visual blind spot. The technical route is illustrated in Figure 3. Firstly, the factors that affect driving safety during VIV are analyzed, including the maximum height of the blind area and the effective sight distance. Then, based on vibration monitoring, a method for the online perception of the dynamic configuration of the main beam is developed. Furthermore, a method for calculating the maximum height and effective distance of the blind spot is provided. The online judgment of these two factors can assist in making some decisions on traffic control. When a long-span suspension bridge experiences VVIV, the framework of online, real-time, and automated perception of blind areas of the bridge deck is started, facilitating real-time traffic safety situation awareness and bridge deck traffic control under VVIV. In the context of an intelligent transportation system, it is an important and concrete example of vehicle–bridge–road collaborative sensing, and it plays a demonstrative role in the development of other intelligent transportation services.

**Figure 4:**
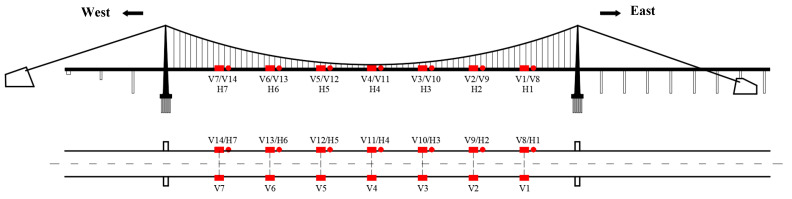
Accelerometer sensor layout on Humen Bridge [23].

Here, we utilize the monitoring data of a VIV event that happened on Humen Bridge [24,25,26] to test the proposed method. The bridge is a single-span suspension bridge with a span of 888 m. The first few natural frequencies of Humen bridge are 0.1344, 0.1705, 0.2325, 0.2768, 0.3687, and 0.4617 Hz according to the test by reference [27]. The monitoring contents of VIV mainly include acceleration, wind speed, and wind direction monitoring. There are seven vertical acceleration sensors (V1–V7, V8–V4) installed on both sides of the main beam, and seven lateral acceleration sensors (H1–H7) installed on one side, as shown in Figure 4. The sampling frequency of the sensor is 50 Hz. The sensor model is 991B, which belongs to a kind of dynamic coil reciprocating vibration pickup. It can measure vibrations as low as 0.072 Hz, with a maximum range of ±15 m/s^2^ and a resolution of 5 × 10^−6^ m/s^2^.

## 3. Online Real-Time Perception and Evaluation of Driving Visual Blind Spots Based on Acceleration Monitoring during VVIV

During this section, monitoring signals from acceleration sensors positioned on the main beam of the suspension bridge experiencing VVIV can be utilized to establish the online perception technical framework of the maximum height of blind areas and the effective sight distance in real time. The following will introduce the fitting of a real-time dynamic configuration of the main beam (which can also be found in our published work [23]; to make this paper as self-contained as possible, we included a description of it in this paper as well) and the theoretical calculation of the maximum height and effective sight distance of the driver’s visual blind area. On this basis, the online perception of driving safety is discussed.

### 3.1. Fitted Real-Time Dynamic Configurations Based on Real-Time Acceleration Integration Algorithm

The monitoring data of multiple vertical acceleration sensors arranged in the main beam are integrated to obtain real-time dynamic displacements of several positions of the main beam; with the help of function fitting, the real-time dynamic configurations with displacements of the measurement points as control points can be made available.

The real-time acceleration integration algorithm proposed by Zheng et al. [28] is used in this paper, which converts the monitoring acceleration data into sampling frames and calculation frames of a specific length, and then the potential baseline is fitted to the calculation frames by using the least-squares and corrected methods. Afterward, a high-pass filter eliminates low-frequency noise, and the data are then integrated into the time domain. These operations are repeated twice to obtain the velocities and displacements of the same length as the sampled frame in turns.

(1) $$y[n]=\frac{1+a}{2}(x[n]-x[n-1])+ay[n-1]$$

where x[n] and y[n] (n=1, 2, 3…) are input and output signals; a is the filter coefficient, a∈(0, 1).

The filter’s transfer function is as follows:

(2) $$H(\omega )=\frac{1+a}{2}*\frac{1-\exp(-i\omega \Delta t)}{1-a*\exp(-i\omega \Delta t)}$$

where Δt is the sampling time interval of acceleration sensors in the structural health monitoring system.

First, the real-time acceleration integration algorithm is adopted to process the monitoring acceleration data of the suspension bridge suffering from VVIV. The structural locking frequency during VVIV is 0.2268 Hz; the structural base frequency is 0.1348 Hz. Multiple attempts are made to determine the filtering frequency $\omega_{c}=0.1\pi$, which is considerably lower than the structural base frequency; recursive filter parameters a=0.99; the amplitude of the transfer function $\left|H(\omega )=0.975\right|$. Under the values of these configuration parameters, the accuracy of the points is the best.

**Figure 5:**
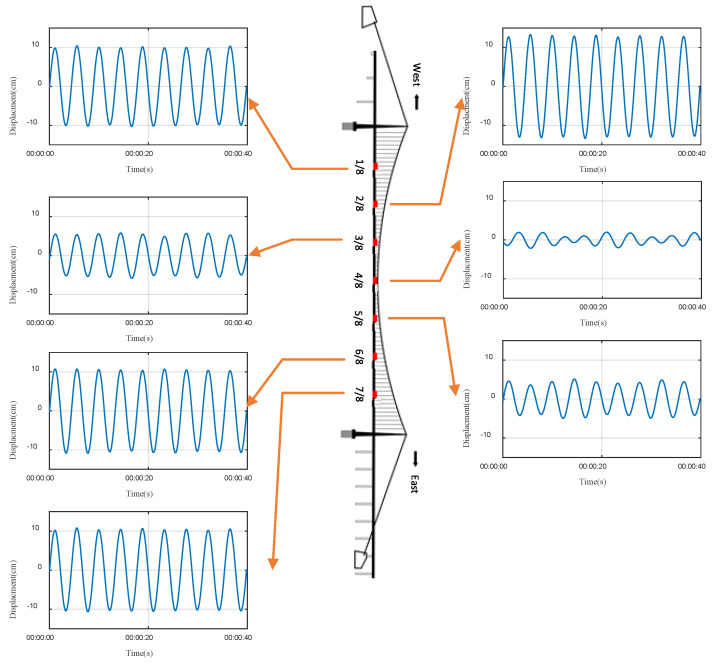
Displacement time history at the eight equal points of the main girder of Humen Bridge.

Figure 5 shows the displacement time history obtained through synchronous online integration of the acceleration signals at the seven measuring points on the main girder during the vortex-induced vibration event of a suspension bridge.

**Figure 6:**
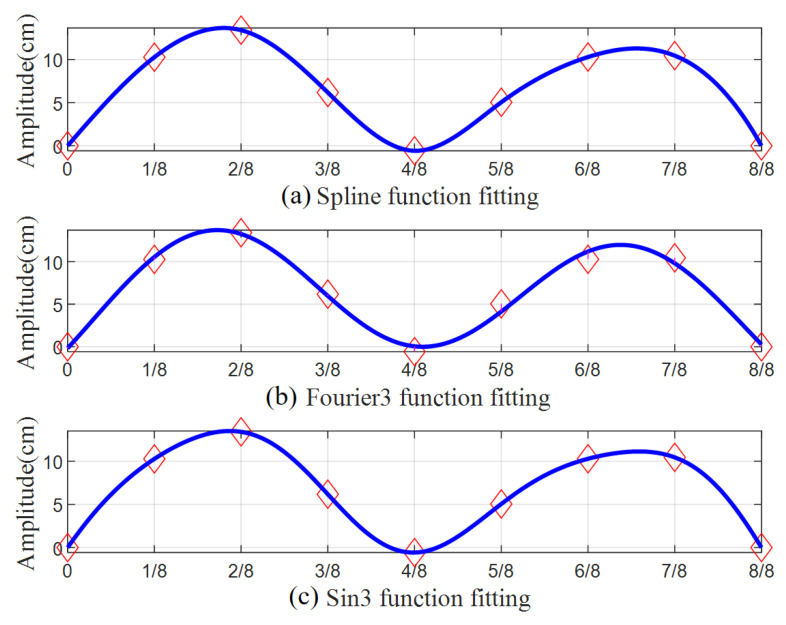
Comparing the fitting effects of three functions (♢ represents data points).

The structural form of Humen Bridge is a single-spanning suspension bridge. In the longitude direction of the bridge, seven sensors are positioned, and seven displacement points can be obtained after integration. There are two hinge fulcrums added; thus, there are a total of nine control points used to fit the configuration of the whole bridge. And three kinds of fitting functions, i.e., spline function, Fourier series, and sin function, are selected. Figure 6 compares the fitting results of these methods. It is found that the Fourier series fitting function does not pass through the control points, but the other two do. And according to the dynamic characteristics of VIV [4,29], we finally select sin function for the fitting because it is more in line with the mode of VIV.

The sine function for fitting is expressed as follows:

(3) $$f(x)=a_{1}\sin(b_{1}x+c_{1})+a_{2}\sin(b_{2}x+c_{2})+a_{3}\sin(b_{3}x+c_{3})$$

**Figure 7:**
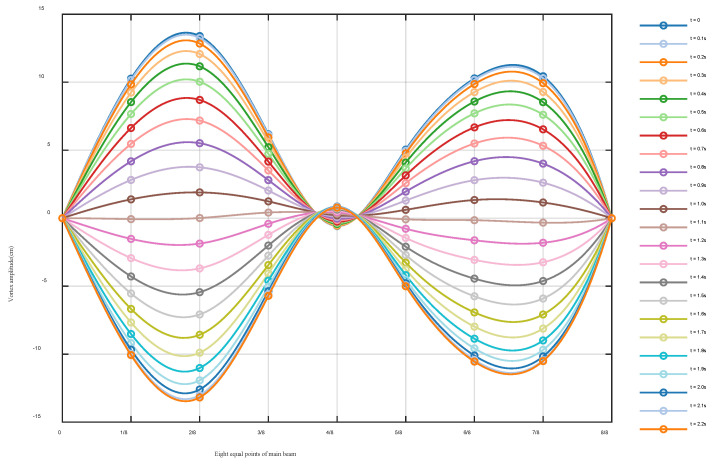
Real-time dynamic configurations of the main beam suffering from VVIV (the time interval is 0.1 s from 0 s to 2.2 s).

With Equation (3) used to fit the data, the real-time dynamic configurations of the main girder can be obtained. Figure 7 shows the evolution process of the dynamic configurations of the main beam for half of the vibration period. After comparing with the finite element results [26,30], the vibration mode of the main girder is the second-order vertical bending symmetry, which is the “M” type, and the vibration period is 4.4 s. This vortex vibration real-time configuration has a maximum displacement of 13.64 cm and a minimum displacement of −13.46 cm. The longitudinal dynamic configurations are not entirely symmetrical to the main beam, and there is a certain amount of hysteresis in the right half. The possible reason is that VIV may occur in the left span first, and then move to the right span.

**Figure 8:**
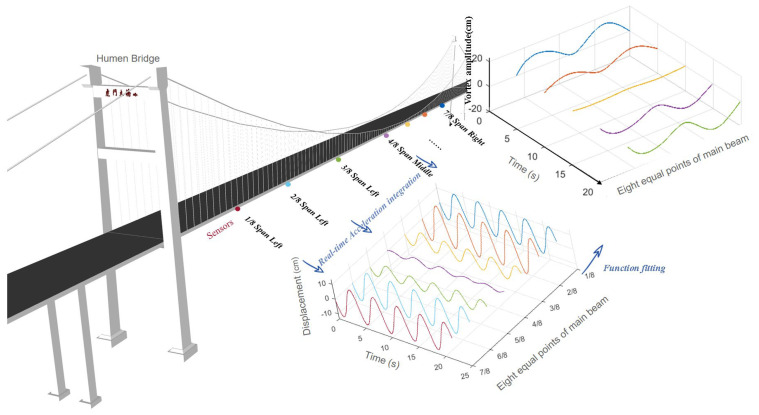
Diagram of real-time dynamic configurations of Humen bridge.

Fitted real-time dynamic configurations of the main beam experiencing VVIV based on a real-time acceleration integration algorithm are shown in Figure 8.

### 3.2. Theoretical Derivation of Driver’s Effective Sight Distance and Maximum Height of Blind Spot

**Figure 9:**
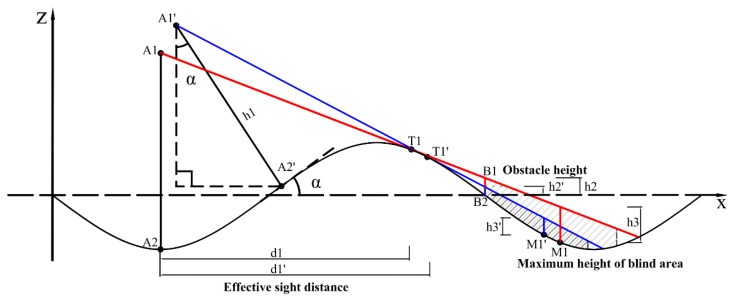
Change in effective sight distance and maximum height of blind area of the driver at positions A2 and A2′ of bridge experiencing VVIV.

As a result of the main girder suffering from VVIV, the bridge deck also undergoes periodic vertical vibrations along the bridge axis. A vortex model shape is a combination of several peaks and troughs. While driving (or parking) on a bridge deck where VVIV occurs, the driver’s view of the adjacent wave trough is most likely obscured by the front wave crest. When a vehicle is located just inside the trough of a bridge deck, its driver has an unfavorable sightline. If there are more than three half-waves in the vibration mode, the wave crests farther away from the driver can also obstruct his sightline; however, the driver pays particular attention to the road conditions that are closer to him in front while driving. As a result, this paper uses the VIV mode comprising three half-waves as an example and develops the driving sightline calculation model based on the dynamic configuration, as shown in Figure 9. Shaded areas in the figure represent the blind spots of the driver as they drive the vehicle over the bridge. Figure 9 clearly illustrates how the vehicle’s visual blind spot changes in response to its movements.

Coordinates of the car (A2′) at time *t* can be expressed as follows:

(4) $$\left\{\begin{array}{l} x_{v}=\dot{u}t\\ z_{v}=v(x_{v},\;t) \end{array}\right.$$

where $\dot{u}$ represents the vehicle’s speed. $x_{v}$ indicates the abscissa of the vehicle on the main beam at the current moment. $z_{v}$ represents the current ordinate of the vehicle on the main girder. v(x,t) indicates the current configuration expression.

The current slope of the tangent line at the car’s position is expressed as follows:

(5) $${\left.\frac{\partial v(x,\;t)}{\partial x}\right|}_{x=x_{v}}=\tan(\alpha )=k$$

At time *t*, at the position of the vehicle, the angle α between the tangent line and the horizontal plane is expressed as follows:

(6) $$\alpha =\arctan\left({\left.\frac{\partial v(x,\;t)}{\partial x}\right|}_{x=x_{v}}\right)$$

Coordinates A1′ of the eye at time t are given by the following:

(7) $$\begin{array}{l} x_{e}=x_{v}+h_{1}\sin(\alpha )\\ z_{e}=z_{v}-h_{1}\cos(\alpha ) \end{array}$$

where $h_{1}$ is the eye height of the driver, namely, the vertical distance from the eye to the horizontal plane.

Assuming the abscissa of the tangent point T1′ is $x_{T}$, then the ordinate $z_{T}$ is given by the following:

(8) $$z_{T}=v(x_{T},\;t)$$

Equating the slope *k* of the sightline equation with the slope of the tangent line to the dynamic configuration equation at that point, we obtain the following:

(9) $$k=\frac{z_{e}-z_{T}}{x_{e}-x_{T}}={\left.\frac{\partial v(x,t)}{\partial x}\right|}_{x=x_{T}}$$

Placing the variable x alone on the left-hand side of the equation gives the following:

(10) $$x=x_{T}=x_{e}-\frac{z_{e}-z_{T}}{{\left.\frac{\partial v(x,t)}{\partial x}\right|}_{x=x_{T}}}$$

Solving this one-variable nonlinear equation gives the result of the abscissa $x_{T}$ of the tangent point. The above equation is a nonlinear equation, solved using iterative methods. When performing continuous calculations, the solution from the previous step can be used as the initial value for the current step, thereby reducing computational complexity.

The effective sight distance is the horizontal distance from the nearest tangent point to the eye. Unless the sightline is tangent to the main beam, the effective sight distance is defined as half of the length of the bridge. According to their driving experience, the driver only catches about half of the bridge when entering the bridge.

The current mathematical expression of the driving sightline is as follows:

(11) $$z(x)=k(x-x_{T})+z_{T}$$

The coordinate B2 $\left(x_{s},\;z_{s}\right)$ of the stationary point is closest to the tangent point between the sightline and the configuration.

(12) $$z_{s}(x_{s})=k(x_{s}-x_{T})+z_{T}$$

The height of the obstacle is assumed to be $h_{2}^{\prime }$:

(13) $$h_{2}^{\prime }=z_{s}(x_{s})$$

In the blind area, the maximum invisible height $h_{3}^{\prime }$ occurs at the tangent point T1′. As shown in Figure 9, the tangent points T1 and T1′ are symmetrical about the stagnation point B2, and $h_{2}^{\prime }$ and $h_{3}^{\prime }$ have a geometric relationship. The maximum height of the blind spot $h_{3}^{\prime }$ is expressed as follows:

(14) $$h_{3}^{\prime }=2h_{2}^{\prime }$$

In the above method, the maximum height $h_{3}^{\prime }$ of the blind zone of the high-order mode can be obtained. However, based on the acceleration monitoring data collected in this paper, the estimation of the main girder’s real-time dynamic configurations (low-order mode) under VVIV is inconsistent with the above-mentioned double relationship. Therefore, in this study, the numerical solution is used to determine the height of the blind spot, i.e., the maximum difference between the sightline equation and the configuration equation of the driver’s visual blind spot.

The blind zone’s maximum height $h_{3}^{\prime }$ at the current time $t_{c}$ is as follows:

(15) $$h_{3}^{\prime }=\max\left(z\left(x,t_{c}\right)-v(x,t_{c})\right)\;x_{T}\leq x\leq x_{J}$$

where $x_{T}$ is the abscissa of the tangent point between the sightline and the configuration; $x_{J}$ is the abscissa of the intersection of the sightline and the configuration.

When the vehicle moves away from the bridge, the sightline has a tangent point with the dynamic configuration of the main beam; however, the extension line does not intersect with the dynamic configuration of the main beam. In this situation, the maximum value of the blind zone height between the main beam’s endpoint and the tangent point is considered the maximum height of the blind area.

### 3.3. Online Perception of Maximum Height of Blind Spot, Effective Sight Distance, and Driving Safety

**Figure 10:**
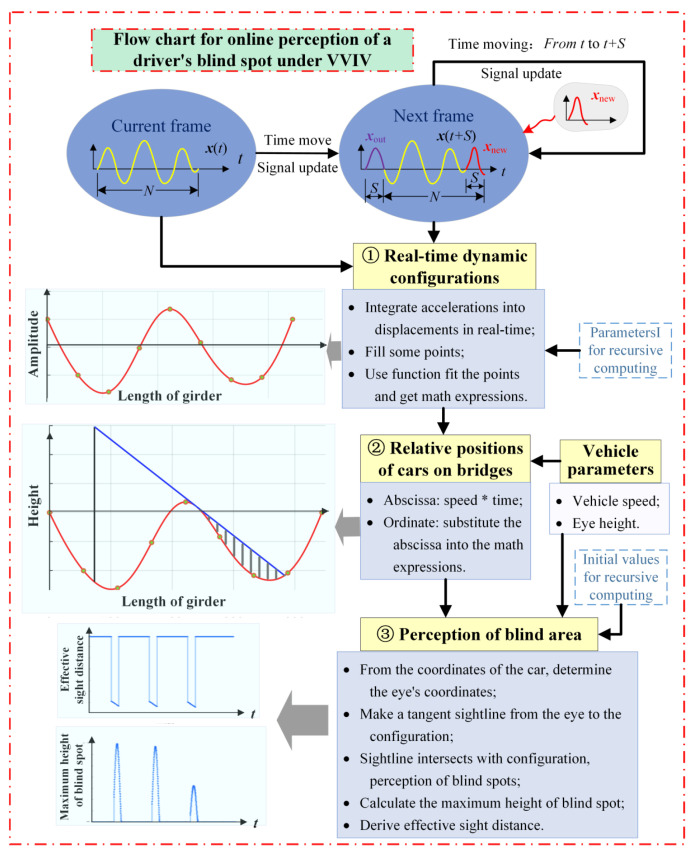
Flow chart of online perception of maximum height and effective sight distance of driver’s visual blind spot on suspension bridges experiencing VVIV.

**Figure 11:**
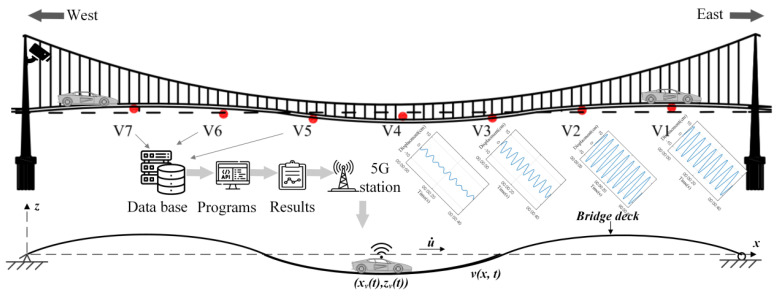
Diagram of vehicle–bridge cooperative sensing under VVIV.

To perceive driving safety online, it is necessary to develop an integrated data processing solution for streaming acceleration monitoring data to complete the calculation of driving blind spots during VVIV. Not only can this data processing solution detect the driver’s far blind spot online in real-time under VVIV, but it can also accurately calculate the parameters related to the far blind spot in real time, including the maximum height of the blind spot and the effective sight distance. Meanwhile, in the proposed scheme, the solution of the equation of the previous frame is used as the initial value of the equation of the next frame, resulting in a more precise and efficient solution. Not only can the framework established in this paper perceive a driver’s far blind area in an online environment, but it can also investigate the change laws of a far blind area in an offline situation. Figure 10 shows the flow chart of this processing scheme, which mainly includes three modules:

(1) Real-time dynamic configuration module: First, the measured acceleration integration algorithm is applied to calculate the dynamic displacements in real time, and the displacement signal can be obtained at the same frequency as the acceleration sampling frequency. For the dynamic displacements of multiple positions on the main beam, the method uses the recursive least squares method to correct the data baseline and recursive high-pass filtering to accurately remove low-frequency noise in the monitoring acceleration signals, and then performs synchronous integration of the acceleration signals. By using these displacements as control points, the dynamic configurations of the main girder are generated through function fitting.

(2) Relative positions of vehicles on bridges: Assuming the time when the vehicle enters the bridge as the starting point, one must input parameters related to the vehicle, such as speed, and then multiply the time to obtain the abscissa of the vehicle on the bridge. By incorporating the abscissa into the dynamic configuration expression obtained in the previous module, the ordinate of the vehicle on the bridge is available. Lastly, the relative positions of the vehicle on the bridge are determined. To perceive the driving blind area online, it is necessary to know the position of the vehicle on the bridge. Through cameras installed on bridge towers, the YOLO method can be used to identify vehicle information, such as vehicle type, speed, and position on the bridge, and then bring them into the frame to determine the driving far blind spot. Or the vehicle perceives its position on the bridge by itself and uses the 5G base station to transmit the real-time road conditions to the vehicle, as shown in Figure 11.

(3) Online perception of driving visual far blind spot module: According to the previous module, one must determine the vehicle’s position on the bridge, deduce the slope of the tangent line at that position, and determine the angle between the tangent line and the horizontal plane. In addition, it is necessary to derive the coordinates of the eye from the eye height. Moreover, one must make a sightline tangent to the configuration from the eye, and when the sightline intersects with the configuration, this causes a visual blind spot. At last, the maximum height of the blind area and the effective sight distance are calculated.

**Figure 12:**
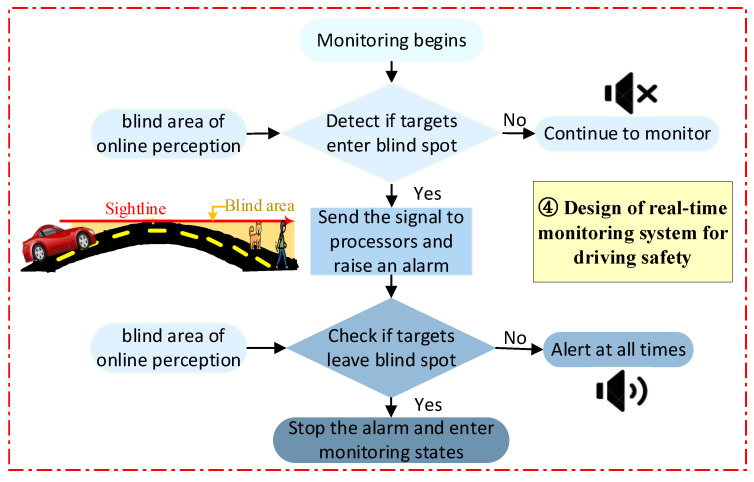
Flow chart of online perception of driving safety on suspension bridges experiencing VVIV.

The framework is capable of perceiving the blind spot of a vehicle under VVIV online. However, this is not enough to ensure driving safety. To better perceive driving safety on the whole bridge during VVIV, the present scheme adds a real-time driving safety online perception module based on the three modules of perception of the blind spot, as shown in Figure 12.

Online perception of driving safety module: When the driving safety monitoring system is used [31], detectable objects (people, cats, dogs, etc.) enter the blind spot of the driver’s visual blind area. The particular wavelength infrared rays emitted by mammals are captured by the sensor, and the sensor activates the alarm module, completing the detection–processing–alarm cycle; if no monitoring object is present in the blind area, the sensor remains silent. With real-time data from the acceleration sensors in the bridge health monitoring system, the blind spot of the driver is perceived online, and the wireless communication method is then transmitted to the vehicle’s intelligent algorithm to judge driving safety, adjust the speed, and initiate automatic driving.

## 4. Online Perception of Maximum Height of Blind Area and Effective Sight Distance According to Vehicle Factors

This part first introduces an online perception of the maximum height of the blind area and the effective sight distance of the car. Then, the effective sight distance and maximum height of the blind spot are perceived online according to the vehicle factors, including vehicle types, vehicle speeds, and different times of vehicles entering the bridge.

### 4.1. Application of the Framework to a Real Bridge

Currently, we only have the measured data of the 14 cm amplitude of Humen Bridge suffering from VVIV. According to real-time dynamic configurations of the monitoring data, the tangent point between the sightline and the dynamic configuration was not detected, and neither were the effective sight distance and maximum height of the blind spot; furthermore, the measured data of the 40 cm amplitude of Humen Bridge experiencing VVIV cannot be obtained for testing. As bridge spanning capacity increases, the vibration amplitude of a suspension bridge with a span of 2000 m will exceed 50 cm or more. The accelerations will be expanded to four times their original values and will then be integrated to determine displacements, i.e., the VVIV amplitude of 0.56 m, which will be used to perceive the effective sight distance and maximum height of the blind spot in the following discussion.

**Figure 13:**
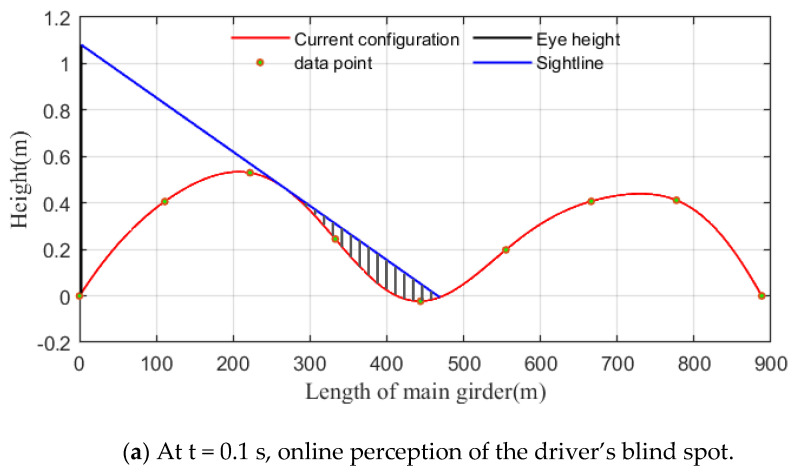

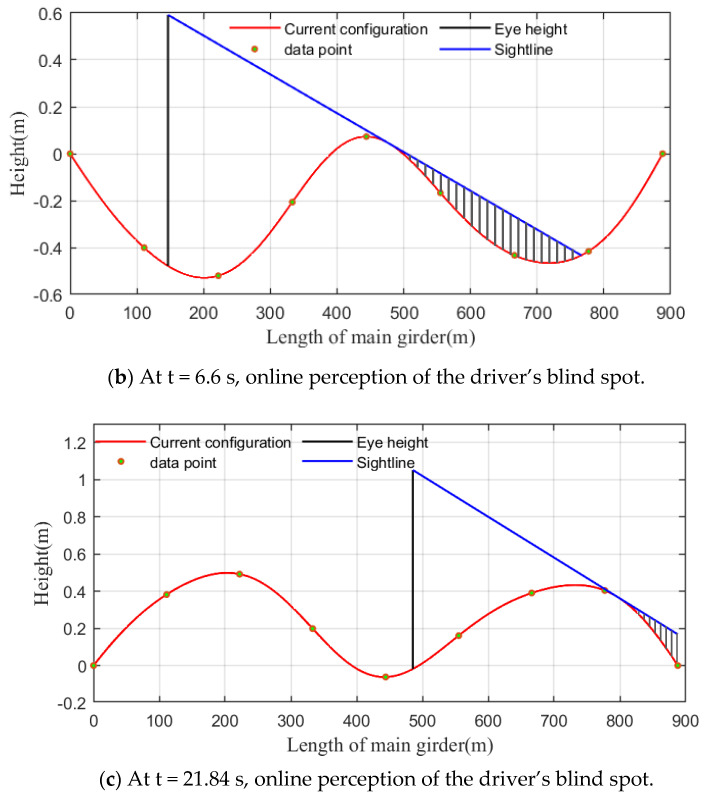
Online perception and changes in the blind spot in the driver’s vision (shaded areas represent far blind spots).

This section takes a car as an example to perceive the driver’s visual blind spot online while the vehicle is moving. The eye height of the driver in the car is 1.07 m. The car with a speed of 80 km/h starts from the bridge, and the timer is set at zero. Based on the data, the vibration amplitude is 56 cm, and the vibration mode is a second-order symmetrical vertical bend which has an “M” shape. The following discussion is based on this mode shape and amplitude, and will not be repeated for other mode shapes and amplitudes. Figure 13 shows the perceived blind spots in the driver’s field of vision at three different moments.

**Figure 14:**
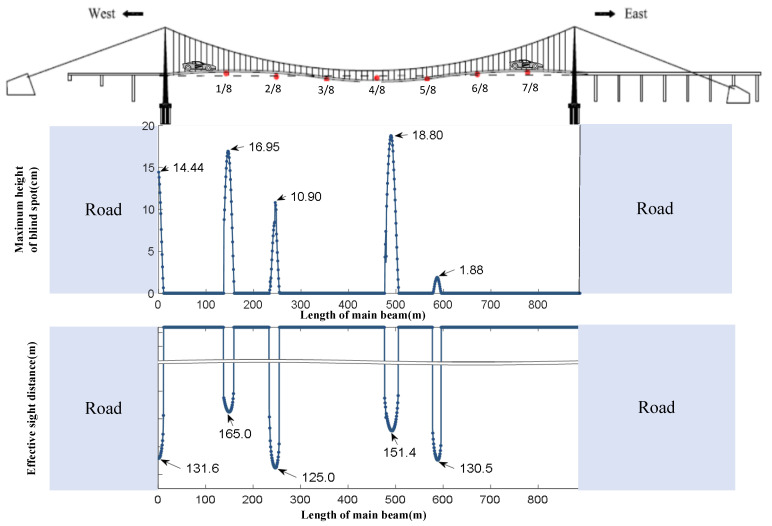
Online perception of the maximum height of the blind spot and the effective sight distance while driving on the entire bridge deck experiencing VVIV. (The vertical axis truncation line in the figure better shows the change in effective sight distance).

The periodic undulating motion of the front bridge deck caused by the VVIV of the main girder can lead the driver to experience periodic blind spots while driving on the bridge. Figure 14 shows that while a vehicle is moving on the bridge where VVIV occurs, the driver’s blind spot appears five times in total, and its location is approximately 0/8, 1/8, 2/8, 4/8, and 5/8 of the main beam. Four blind spots have a maximum height of more than 10 cm, and the maximum height is 18.80 cm near 4/8 of the main beam. As the effective sight distance changes suddenly at these locations, five times in total, the minimum distance is 125.0 m, near 2/8 of the main beam, which is where the driver’s sightline is the worst. The maximum height of the blind spot and the sightline distance are not continuous over time because the adjacent half-waves are too low to obstruct the driver’s vision when the vehicle travels normally on a bridge experiencing VVIV. At this time, the maximum height of the blind spot is 0, and the effective sight distance is a half wavelength.

### 4.2. Online Perception of Maximum Height of Blind Area and Effective Sight Distance with Different Vehicle Models

During this section, the vehicle speed is 80 km/h, and the drivers of different models are driving on a bridge experiencing VVIV; these criteria are used to perceive the effective sight distance and maximum height of the blind spot online. Four types of vehicles are considered: cars, SUVs, vans, and large container vehicles. The drivers’ eye height of the five vehicle models are shown in Table 1 based on the data.

**Figure 15:**
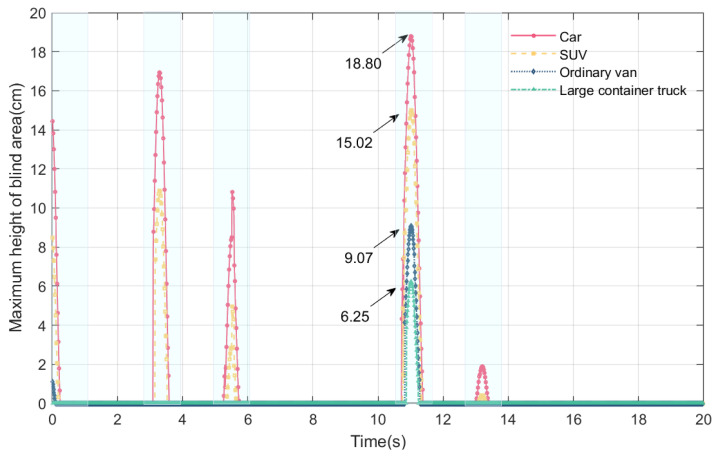
Online perception of maximum height of blind spot of different vehicle models during VVIV.

**Figure 16:**
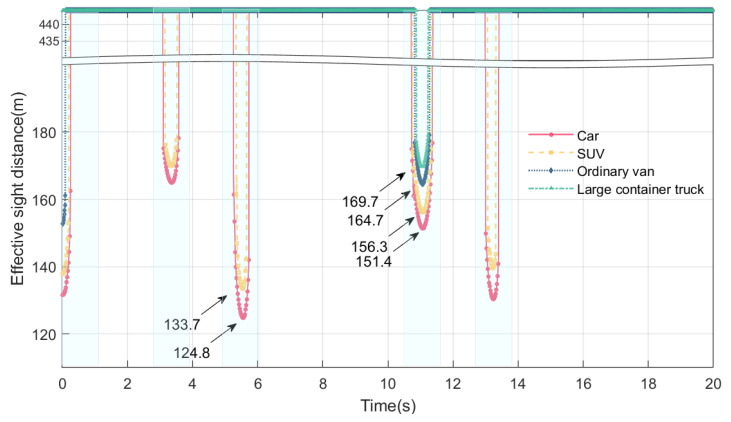
Online perception of effective distance of different vehicle models during VVIV.

From Figure 15 and Figure 16, it can be seen that when the vehicle is moving on the bridge, the maximum height of the blind spot and the effective sight distance of the driver are subject to periodic changes. The maximum height and effective sight distance of the blind spot are different for different models. The maximum height of the driver’s blind spot and the minimum effective sight distance of the five models are shown in Table 2. The worst sightline is in cars, followed by SUVs, vans, and large container trucks. The duration of the blind spot varies from vehicle to vehicle. The blind spot duration of cars is the longest, large container trucks have the shortest, and vans and SUVs are in the middle.

A driver’s eye height is mainly responsible for the difference between the maximum height of the driver’s blind spot and the effective distance in different vehicle models. In a car, the apparent height of the driver is the lowest among the five models, at 1.07 m, resulting in the largest visual blind spot and the shortest effective sight distance. The lower the driver’s eye height is, the higher the maximum height of the driver’s visual blind spot will be, and the shorter the effective sight distance will be. Therefore, the driver’s sightline in the car is the worst when the bridge deck experiences VVIV, while the driver’s sightline in the large container truck is better than in the other three vehicles. Due to the downward deflection of the main beam caused by the vehicle’s weight, the height of the driver’s blind spot will be increased further. Qualitatively speaking, since the large container truck is heavier, causing a certain amount of downward deflection of the main beam, this results in a worse driving sightline for the large container truck. Among the four models, the driver’s sightline in the car is the worst, and the driver’s sightline in the large container truck is the best during VVIV.

### 4.3. Online Perception of Maximum Height of Blind Area and Effective Sight Distance at Different Vehicle Speeds

During the service period of Humen Bridge, the daily traffic volume is large, and the standard speed of the main bridge is increased from 80 km/h to 100 km/h. At different speeds of three vehicles, namely, 80 km/h, 90 km/h, and 100 km/h, the maximum height of the driver’s visual blind spot and the effective sight distance in the car are perceived online in real time. The vehicle type used in this section is the car.

**Figure 17:**
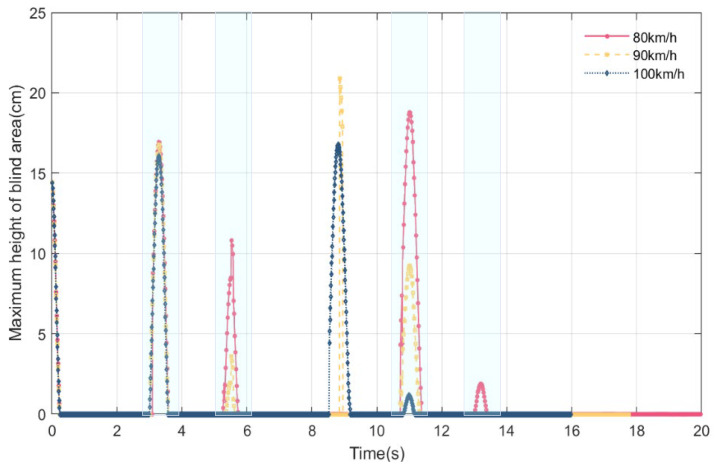
Online perception of maximum height of blind area at different vehicle speeds during VVIV.

**Figure 18:**
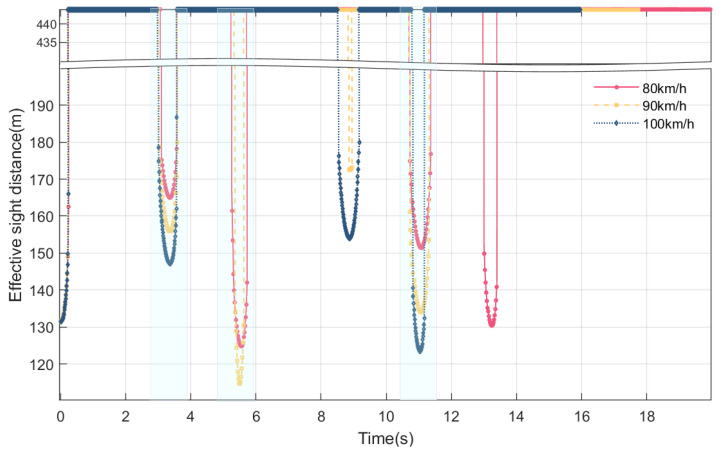
Online perception of effective sight distance at different vehicle speeds during VVIV.

As is apparent from Figure 17 and Figure 18, in terms of the duration of the blind spot, as the vehicle’s speed increases, the maximum height of the blind spot decreases, and the effective sight distance decreases. When the vehicle travels at 80 km/h, the blind spots appear at around 0, 3.28 s, 5.22 s, 11.01 s, and 13.18 s. As the vehicle speeds up to 90 km/h, the maximum height of the blind spot at these times decreases, and the effective sight distance increases; however, a blind spot is visible at a speed of 90 km/h around 8.86 s. As the vehicle speeds up to 100 km/h, the maximum height of the blind spot at these times decreases or even disappears, and the effective sight distance increases. As the vehicle’s speed increases from 80 km/h to 100 km/h, the number of occurrences of visual blind spots decrease, and the total duration of the blind spots decreases. In general, as the vehicle’s speed increases, when the previous blind spot reappears, the maximum height of the blind spot decreases or even disappears, and the effective sight distance increases.

### 4.4. Online Perception of Maximum Height of Blind Area and Effective Sight Distance at Different Times of Vehicle Entry onto the Bridge

In this section, we discuss the online perception of the effective sight distance and the maximum height of the blind spot when a vehicle enters the bridge at different moments, and the influence of the initial vibration configuration of the main beam on the maximum height of the blind spot and the effective sight distance. We select the time of vehicle entry onto the bridge within a vibration period; the vibration peak of the main beam is the largest, followed by a vibration peak of 0, then the vibration trough is the largest, and finally, the vibration trough is 0. Taking a car driving in VVIV mode at a speed of 80 km/h as an example, we show the results of the maximum height of the driver’s blind spot and the effective sight distance at different times of vehicles entering the bridge.

**Figure 19:**
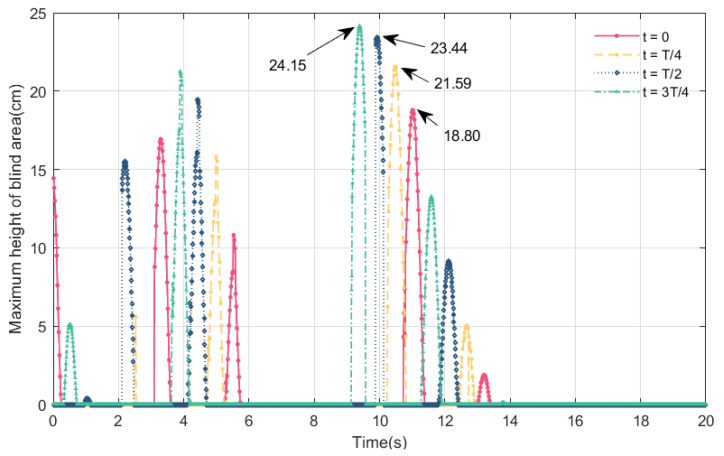
Online perception of maximum height of blind area at different times of vehicles entering bridges experiencing VVIV.

**Figure 20:**
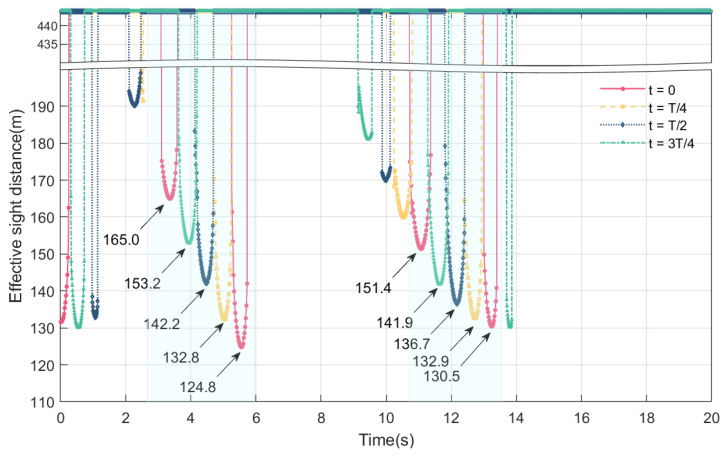
Online perception of effective sight distance at different times of vehicles entering bridges experiencing VVIV.

From Figure 19 and Figure 20, it can be observed that the maximum height of the blind spot and the effective sight distance change periodically at different times of vehicles entering the bridge at the same speed. At different times of vehicles entering the bridge, the visual blind spot appears twice in 9 s~13.5 s, and its maximum height is the largest during the driving process. Compared with other moments of the vehicle entering the bridge, such as when the trough is 0, the maximum height of the blind spot is 24.15 cm. The order of the effective sight distance changes in that the peak is the largest, the trough is 0, then the trough is the largest, and the peak is 0. The minimum effective sight distance is 124.8 m.

Based on the data in Table 3, it can be seen that the vehicle enters the bridge when the peak of the wave is the largest, the number of occurrences of the visual blind spot are the highest and the maximum height of the blind spot exceeds 10 cm; moreover, when the wave peak is 0, the number of blind spots are fewer, the maximum height of the blind area is less than 10 cm, and the blind spot duration is the shortest. Drivers who enter the bridge at the wave’s peak have the smallest effective sight distance, while those who enter at the largest trough have the largest effective sight distance. As a result, when the vehicle enters the bridge at the peak of 0, that is, when the bridge deck is from the peak of 0 to the maximum trough, there are fewer blind spots, thus contributing to driving safety.

This section mainly discusses the online perception and change law of the maximum height of the blind spot and the effective sight distance under different conditions, including the time of the vehicle entering the bridge, vehicle types, and speeds. Among them, the main factor affecting the maximum height of the blind spot and the effective sight distance is vehicle type, followed by vehicle speed and the moment when the vehicle enters the bridge. Specifically, the lower the driver’s eye height in the car is, the smaller the effective sight distance will be, and the higher the height of the driver’s visual blind spot will be; as vehicle speed increases, the maximum height of the blind spot decreases, as does the effective sight distance. If the vehicle enters the bridge when the vibration peak is 0, the duration of blind spot is short, and the maximum height of the blind spot is small.

The results of this paper mainly rely on the second-order VVIV mode of Humen Bridge with a span of 888 m; if the higher-order mode is encountered, the number of blind spots will increase, the effective sight distance will reduce, and the duration of the blind zones will be longer; in the case of bridges with a greater span, the maximum height of the blind spot and the effective sight distance are more periodic, and the blind spot lasts longer. Various factors influence the blind spot; however, when several factors are combined, which is the most unfavorable situation, it is necessary to pay attention to the change in the blind spot. For example, in the case of a high-order mode shape, large amplitude, steep longitudinal slope, and driving into a VVIV peak at a high speed, great attention has to be paid to the visual blind spot to ensure driving safety. When traveling on a bridge experiencing the small amplitude of VVIV, the driver should not panic, and should maintain a safe distance from the vehicle in front and proceed as usual. When long-term and excessive amplitude of VVIV occurs on the bridge, the transportation department should take measures to limit vehicles’ orderly passage and temporarily shut down the lanes.

## 5. Conclusions

This paper presents for the first time an online perception framework for estimating the maximum height and the effective sight distance of the far blind spot when traveling on a long-span suspension bridge suffering from VVIV. On this basis, it could offer reasonable suggestions for traffic control in terms of driving safety. The proposed method is also suitable for other types of bridges that may experience VVIV.

On the bridge where VVIV occurs, the driver will experience periodic changes in their visual blind spot. The vehicle type has the most significant effect on the maximum height of the blind spot and on the effective sight distance. As the vehicle’s speed increases, the number of blind spots decrease, as well as the values of the maximum height and the effective sight distance.

By analyzing the real-time data of the acceleration sensor in the bridge health monitoring system, the driver’s visual blind spot can be perceived online. Through wireless communication, the evaluation results of the blind spot can be transmitted to the vehicle’s intelligent algorithm, which judges driving safety and adjusts the vehicle’s speed accordingly. Therefore, it can provide a specific application scenario of bridge–vehicle collaborative perception for intelligent transportation.

## Figures and Tables

**Table 1:** Parameters related to drivers’ sightline.

Vehicle Model	Car	SUV	Ordinary Van	Large Container Truck
Eye height: h1 (m)	1.07	1.2	1.45	1.6

**Table 2:** Comparison of the blind spot and effective sight distance of different vehicle models.

Vehicle Models	Car	SUV	Ordinary Van	Large Container Truck
Number of blind areas	5	5	2	1
Maximum height of blind areas (cm)	18.80	15.02	9.07	6.25
Effective sight distance (m)	124.8	133.7	164.7	169.7

**Table 3:** Comparison of results of different times of vehicles entering bridges.

Different Times of Vehicles Entering Bridges	The Peak of the Wave Is the Largest	The Peak of the Wave Is 0	The Trough of the Wave Is the Largest	The Trough of the Wave Is 0
Number of blind areas	5	4	4	5
Maximum height of blind area over 10 cm	4	2	3	3
Minimum effective sight distance at different times (m)	124.8	132.8	136.7	130.5

## Data Availability

The data presented in this study are available on request from the corresponding author due to privacy.
